# Green and Energy-Efficient Post-Harvest Enhancement: Low-Dose ^60^Co-*γ* Irradiation Enhances the Bioactive Profile of Lily Bulbs (*Lilium lancifolium* Thunb.) as Functional Food Ingredients

**DOI:** 10.3390/antiox15091071

**Published:** 2026-08-26

**Authors:** Yao Hu, Lihui Li, Xingyu Lei, Yiji Zhou, Wei Gong, Mengshan Sun, Rong Song

**Affiliations:** Hunan Institute of Nuclear Agriculture Sciences and Chinese Herbal Medicines, Hunan Academy of Agricultural Sciences, Changsha 410125, China

**Keywords:** lily bulbs, low-dose irradiation, post-harvest enhancement, metabolomics, antioxidant activity, molecular docking

## Abstract

Edible lily bulbs (*Lilium lancifolium* Thunb.) are highly valued for their rich nutritional profile and significant medicinal properties. To further enhance these beneficial attributes, this study investigates a novel, green post-harvest enhancement strategy using low-dose ^60^Co-*γ* irradiation (2–5 Gy) followed by immediate freeze-drying. Experimentally, fresh bulbs were exposed to varying irradiation doses, after which physiological parameters (enzyme activities, phytochemical contents) and metabolic profiles (via LC-MS/MS) were systematically evaluated. The results demonstrated that 4 Gy represented the optimal dose; compared to the non-irradiated control group (0 Gy), the 4 Gy treatment significantly enhanced the antioxidant defense system by eliciting oxidative stress responses, increasing superoxide dismutase (SOD) and catalase (CAT) activities by 1.55- to 1.86-fold, and elevating total phenolics and flavonoids by 1.31- and 2.69-fold, respectively. Untargeted metabolomic analysis identified 42 differential metabolites, revealing that 4 Gy irradiation primarily upregulated the phenylpropanoid and flavonoid biosynthetic pathways. Key antioxidants, specifically rosmarinic acid and the newly detected alkaloid jurubine, exhibited significant accumulation. Integrated correlation analysis and molecular docking simulations elucidated the underlying mechanisms, providing theoretical insights into their transient interactions with DPPH radicals. In conclusion, low-dose ^60^Co-*γ* irradiation serves as a sustainable, green post-harvest processing strategy to improve the nutritional quality of edible lily bulbs by activating intrinsic metabolic defense mechanisms.

## 1. Introduction

The genus *Lilium* comprises numerous species that are globally renowned for their aesthetically pleasing flowers and hold immense commercial importance in the cut flower industry [1,2]. It is important to note that many are cultivated strictly for ornamental purposes, and some species are non-edible and can be toxic to humans and animals due to the presence of harmful alkaloids. In contrast, a select few species, notably *Lilium lancifolium* Thunb., have a long history of safe consumption and are officially recognized as dual-purpose resources for both food and medicine. Specifically, the edible bulbs of *L. lancifolium* are widely utilized in traditional culinary and functional food applications due to their rich reservoir of bioactive secondary metabolites. These include phenolic acids, flavonoids, saponins, and alkaloids, which exhibit potent antioxidant and immunomodulatory properties [3,4]. With the escalating consumer demand for natural health products, there is a critical need for high-quality, processed lily-based ingredients, such as freeze-dried powders. However, maximizing the retention and bioactivity of these functional phytochemicals during raw material preparation remains a significant industrial challenge.

Conventionally, processing methods rely on thermal treatments, which inevitably degrade heat-sensitive nutrients, or chemical treatments that incite safety concerns regarding residues [5]. While irradiation technology has been applied to lily bulb processing, it predominantly employs high-dose radiation (kGy level) to extend shelf-life by inhibiting physiological metabolism and sprouting [6,7]. Notably, such high-dose treatments are energy-intensive and fundamentally focus on “suppression” for preservation rather than “activation” for metabolic enhancement. Consequently, the industry is actively seeking green, non-thermal technologies capable of enhancing the phytochemical profile of raw materials. Among potential candidates, ionizing radiation (e.g., ^60^Co-*γ*) poses a unique advantage due to its high penetration power and residue-free nature, aligning perfectly with sustainable processing principles [8,9].

Low-dose ^60^Co-*γ* irradiation (Gy level) presents a promising [10], yet underexplored, avenue for quality improvement. Unlike high-dose exposure, low-dose irradiation is cost-effective and functions as an abiotic stressor to induce hormesis—a phenomenon where low-level stress stimulates beneficial biological responses and metabolic adaptations [10]. Scientific evidence suggests that lily bulbs are highly radiosensitive, with a physiological response threshold typically falling between 2 and 6 Gy [11,12,13]. Mechanistically, at the cellular level, this low-dose radiation induces the radiolysis of intracellular water, generating a transient burst of reactive oxygen species (ROS). Rather than causing lethal oxidative damage, this mild ROS accumulation acts as a signal cascade that upregulates defense-related gene expression and antioxidant systems [14]. This implies that a controlled “micro-dose” (2–5 Gy) could sufficiently activate intrinsic biosynthetic pathways, yielding “premium raw materials” enriched with functional compounds. Although previous studies have documented increased phenolics in various crops following irradiation [10,15], research on lilies has primarily focused on storage parameters or heritable breeding traits [6,7,11]. To date, no study has systematically investigated low-dose ^60^Co-*γ* irradiation as a targeted pre-treatment for immediate bioactive enrichment in edible lily bulbs nor elucidated the underlying metabolic mechanisms driving these changes.

Therefore, this study investigates the application of low-dose ^60^Co-γ irradiation (2–5 Gy) to fresh lily bulbs as a novel, sustainable post-harvest metabolic enhancement strategy. To accurately evaluate the authentic irradiation-induced metabolic responses and prevent any secondary thermal degradation of bioactive compounds that typically occurs with conventional drying methods, immediate vacuum freeze-drying was strictly employed for sample preparation. This technique ensures the maximum retention of transient irradiation-induced metabolites by stopping deterioration reactions at low temperatures [16]. The primary objective was to validate the feasibility of this method for nutritional quality improvement. Specifically, we hypothesized that the radiation-induced accumulation of specific bioactive metabolites contributes to the enhanced bioactivity of lily bulbs. By integrating UHPLC-Q-TOF/MS-based metabolomics with molecular docking simulations, we systematically investigated the impact of 2–5 Gy irradiation on the accumulation of bioactive metabolites and antioxidant capacity to verify this hypothesis. This work aims to provide a theoretical basis and technical support for a green, energy-efficient approach to producing superior lily-based functional ingredients.

## 2. Materials and Methods

### 2.1. Chemicals and Reagents

A total of twenty-nine authentic standards for primary metabolites and thirty-five for secondary metabolites were obtained from Sigma-Aldrich (St. Louis, MO, USA); detailed purchase information is provided in the Supplementary Material. HPLC-grade formic acid and acetonitrile were purchased from Merck (Darmstadt, Germany). Deionized ultrapure water (18.2 MΩ·cm) was generated using a Millipore water purification system (Milli-Q Direct 8, Millipore, Bedford, MA, USA).

### 2.2. Material Preparation and Treatment

Fresh lily bulbs (*Lilium lancifolium* Thunb.) were sourced from the Yinjiajie Lily Base, a large-scale commercial farm located in Longshan County, Hunan Province, China. The bulbs were planted in September 2024 and harvested in August 2025, corresponding to a growth cycle of approximately 11 months. Harvesting was conducted at full commercial maturity, which was phenotypically indicated by the yellowing and withering of over two-thirds of the above-ground stem and foliage. The botanical identity of the selected variety was formally authenticated by Prof. Xiaoqi Zhu from the Hunan Research Center of Medicinal Plants. The authentication was based on typical morphological characteristics of the species, including the presence of purplish-black bulbils in the leaf axils, strongly reflexed orange-red tepals, and purple-striped stems. To ensure experimental consistency, bulbs were rigorously screened for uniform size (weighing on average 50.0 ± 5.0 g and measuring 5.0 ± 0.5 cm in diameter) and the absence of mechanical damage, pests, or diseases. The selected samples were transported via a cold chain system (insulated containers with ice packs) to the Irradiation Center at the Hunan Institute of Nuclear Agriculture Sciences and Chinese Herbal Medicines, Hunan Academy of Agricultural Sciences.

**Figure 1:**
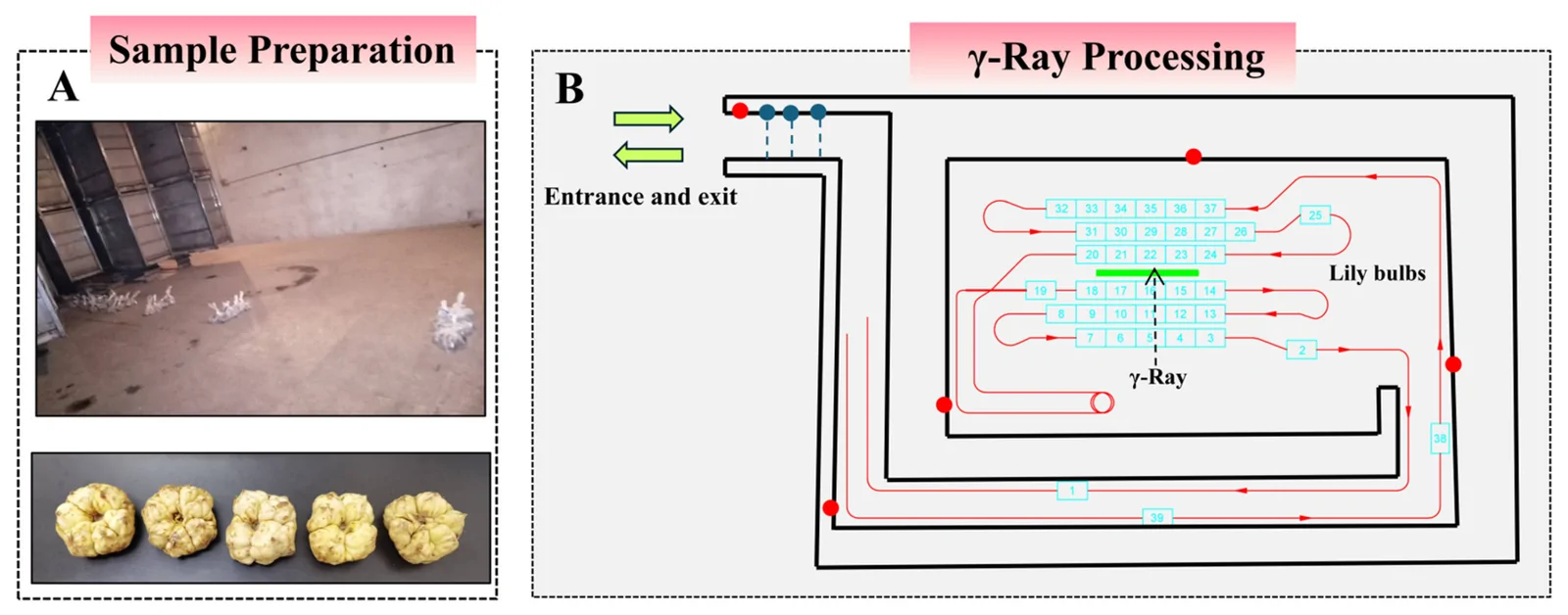
Schematic diagram illustrating the sample preparation and ^60^Co-*γ* irradiation treatment of lily bulbs. (**A**) Sample preparation workflow; (**B**) γ-ray irradiation process. In panel (**B**), the red lines with arrows indicate the running direction of the conveyor belt. The light blue boxes represent the irradiation cargo boxes containing the samples. The central green bar represents the ^60^Co-*γ* radiation source.

The bulbs were randomly assigned to five groups (*n* = 10 per group). Irradiation was performed using an SQ(H) industrial ^60^Co-*γ* irradiation facility (Beijing Sanqiang Nuclear Radiation Engineering Technology Co., Ltd., Beijing, China) with a radioactivity of 3.14 × 10^16^ Bq. Samples were subjected to static irradiation at ambient temperature with target doses of 0 (Control), 2, 3, 4, and 5 Gy. The irradiation treatments were conducted at dose rates of 22, 35, 45, and 55 Gy/h, respectively (the irradiation process is illustrated in Figure 1). Immediately following treatment, samples were vacuum freeze-dried using a vacuum freeze dryer (Model FD-1A-50, Boyikang, Beijing, China) to terminate physiological metabolism and preserve bioactive components. To ensure representative sampling and generate biological replicates (*n* = 3) for each group, the 10 dried bulbs were randomly divided into three sub-samples (comprising 3–4 bulbs each). The bulbs within each sub-sample were then pooled and thoroughly homogenized. All subsequent chemical extractions, antioxidant capacity assays, and metabolomic analyses were performed using uniform aliquots of these homogenized powders, which were stored at −80 °C until further analysis.

### 2.3. Determination of Total Phenolic and Total Flavonoid Contents

For the determination of phytochemical and antioxidant activities, a standardized extraction process was performed. Briefly, 1.0 g of the pooled lyophilized lily bulb powder was extracted with 20 mL of 70% ethanol under ultrasonication for 30 min at room temperature. The mixture was centrifuged at 8000× *g* for 10 min, and the supernatant was collected. The extraction was repeated once, and the combined supernatants were adjusted to a final volume of 50 mL with 70% ethanol. The total phenolic content (TPC) and total flavonoid content (TFC) were quantified according to the methods described by Meng et al. [17], with minor modifications. TPC was determined using the Folin–Ciocalteu colorimetric assay, with results expressed as gallic acid equivalents (GAEs). TFC was measured using the aluminum chloride colorimetric assay, with results expressed as rutin equivalents (REs). Absorbance was recorded using a UV-Vis spectrophotometer (Model UV-1800, Shimadzu, Kyoto, Japan).

### 2.4. Determination of Antioxidant Capacity

Antioxidant capacities of the aforementioned extracts were evaluated using four complementary assays: 2,2′-azino-bis (3-ethylbenzothiazoline-6-sulfonic acid) (ABTS) radical scavenging activity, hydroxyl radical (·OH) scavenging activity, 2,2-diphenyl-1-picrylhydrazyl (DPPH) radical scavenging activity, and total antioxidant capacity (T-AOC). The DPPH and ABTS assays were conducted following the protocols of Wang et al. [7], while the ·OH and T-AOC assays were performed according to Sun et al. [18]. The results for DPPH, ABTS, and ·OH radical scavenging activities were calculated and expressed as the percentage of inhibition (%), whereas the T-AOC was expressed as micrograms per gram of dry weight (U/g DW).

### 2.5. Determination of Antioxidant Enzyme Activities

The activities of superoxide dismutase (SOD), catalase (CAT), polyphenol oxidase (PPO), and peroxidase (POD) were determined using commercial assay kits (Nanjing Jiancheng Biotechnology Research Institute Co., Ltd., Nanjing, China), following the manufacturer’s instructions and the protocol described by Ji et al. [6]. Briefly, 0.1 g of fresh lily bulb tissue was homogenized in 1 mL of the extraction buffer provided in the respective kits using a tissue homogenizer (Model T10 basic, IKA, Staufen, Germany). The homogenate was centrifuged at 10,000× *g* for 10 min at 4 °C using a refrigerated centrifuge (Model 5424R, Eppendorf, Hamburg, Germany), and the supernatant was collected for the enzymatic activity assays.

### 2.6. Metabolomic Analysis

Sample preparation was conducted based on our previous protocols [19,20] with minor adjustments. Briefly, 100 mg of lily bulb powder was extracted with 10 mL of pre-warmed 70% (*v*/*v*) methanol.

Metabolomic profiling was performed using a UHPLC-Q-Exactive Orbitrap mass spectrometer. Chromatographic separation was achieved on an Acquity UPLC HSS T3 column (2.1 mm × 100 mm, 1.8 μm; Waters, Manchester, UK) maintained at 40 °C. The mobile phase consisted of 0.1% (*v*/*v*) formic acid in deionized water (A) and 0.1% (*v*/*v*) formic acid in acetonitrile (B), delivered at a flow rate of 0.4 mL/min with an injection volume of 3 μL. The gradient elution program was as follows: 0–0.5 min, 2% B; 0.5–8 min, 2–15% B; 8–13 min, 15–35% B; 13–15 min, 35–70% B; 15–16 min, 70–85% B; and 16–16.5 min, 85–2% B. Equilibration at 2% B for 8.5 min followed. To minimize instrumental drift, samples were analyzed in a randomized order. Quality control (QC) samples were interspersed throughout the sequence to monitor system stability. Mass spectrometric detection was carried out on a Q Exactive Orbitrap mass spectrometer (Thermo Fisher Scientific, Bremen, Germany) equipped with an electrospray ionization (ESI) source operating in both positive and negative modes. Key parameters were set as follows: capillary voltage, 3.5 kV (positive) and −3.0 kV (negative); capillary temperature, 300 °C; sheath gas flow rate, 10 L/min; and auxiliary gas heater temperature, 325 °C. The mass scanning range was *m/z* 80–1200.

Raw data processing is detailed in the Supplementary Material, according to our previous procedure [19,20].

### 2.7. Molecular Docking Analysis

In silico molecular docking simulations were conducted to investigate the binding mechanisms of rosmarinic acid (CID: 5281792) and jurubine (CID: 52931419) with the DPPH radical (CID: 2735032). Three-dimensional (3D) structures of the ligands and the target radical were retrieved from the PubChem database.

Prior to docking, structures were pre-processed using AutoDockTools (v. 1.5.7, The Scripps Research Institute, La Jolla, CA, USA) to remove water molecules, add polar hydrogens, compute Gasteiger charges, and define rotatable bonds. Docking simulations were executed using AutoDock Vina (v. 1.1.2, The Scripps Research Institute, La Jolla, CA, USA). The conformation exhibiting the lowest binding free energy was selected as the optimal binding pose. Interactions were visualized and analyzed using PyMOL 2.6 (Schrödinger, LLC, New York, NY, USA).

### 2.8. Statistical and Data Analysis

Multivariate statistical analyses, including Principal Component Analysis (PCA) and Partial Least Squares Discriminant Analysis (PLS-DA), were performed using SIMCA 14.1 (Umetrics, Umeå, Sweden). Univariate statistical comparisons were conducted using SPSS 21 (IBM, Chicago, IL, USA) via one-way ANOVA followed by Duncan’s multiple range test. Hierarchical clustering heatmaps were generated using MultiExperiment Viewer 4.9.0 (Dana-Farber Cancer Institute, Boston, MA, USA) with unit variance (UV) scaling normalization. The correlation network between antioxidant indices and differential metabolites was constructed and visualized using Cytoscape (version 3.9.1, Cytoscape Consortium, San Diego, CA, USA).

## 3. Results and Discussion

### 3.1. Impact of Irradiation on Antioxidant Properties

**Figure 2:**
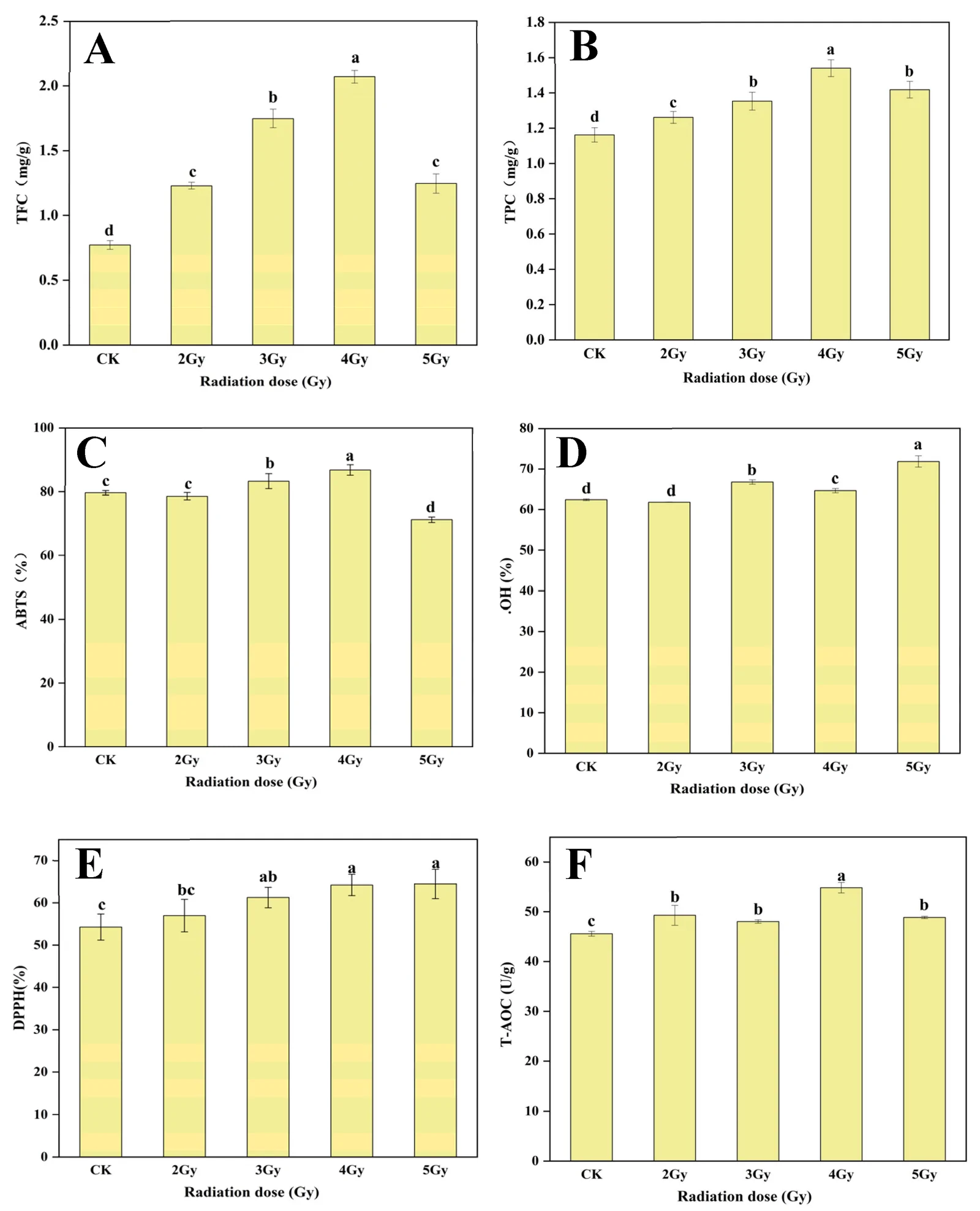
Effects of irradiation treatment on the contents of total flavonoids (TFC) and total phenolics (TPC), and antioxidant activities in lily bulb extracts. (**A**) TFC (mg/g DW); (**B**) TPC (mg/g DW); (**C**) ABTS radical scavenging activity; (**D**) ·OH scavenging activity; (**E**) DPPH radical scavenging activity; (**F**) total antioxidant capacity (T-AOC). Data are presented as mean ± SD. Different letters indicate statistically significant differences among treatments (*p* < 0.05).

Phenolic compounds and flavonoids are the predominant bioactive constituents in plants, renowned for their potent antioxidant and free radical scavenging capabilities [3]. As shown in Figure 2A,B, low-dose irradiation exerted a significant, dose-dependent influence on the accumulation of these secondary metabolites. Specifically, the total phenolic content (TPC) in the 3 Gy and 4 Gy groups increased by 1.16- and 1.31-fold, respectively, compared to the control (CK) group (*p* < 0.05). However, a marked decline was observed when the dose was increased to 5 Gy. Similarly, the total flavonoid content (TFC) exhibited a characteristic bell-shaped trend, peaking at 4 Gy with a 2.69-fold increase over the control. These results align with previous reports [7,21] and can be explained satisfactorily by the phenomenon of radiation hormesis. This phenomenon suggests that low-dose stress acts as a stimulant for the phenylpropanoid metabolic pathway, triggering the de novo synthesis of antioxidants as a defensive mechanism. Additionally, irradiation may facilitate the release of bound phenolics by partially disrupting cellular wall structures, thereby increasing extractability [7,22].

To comprehensively evaluate bioactivity, four distinct assays were employed: ABTS, ·OH, DPPH, and T-AOC. As illustrated in Figure 2C,F, while the 2 Gy treatment showed no significant difference from CK (*p* > 0.05), doses ranging from 3 to 5 Gy significantly enhanced radical scavenging capacities (*p* < 0.05). Notably, the total antioxidant capacity (T-AOC) reached its maximum at 4 Gy (1.24 times that of CK). The striking synchronization between metabolite accumulation and antioxidant capacity confirms that 4 Gy serves as an optimal “activation” threshold for the phytochemical enrichment of lily bulbs.

### 3.2. Response of Antioxidant Enzyme Activities

**Figure 3:**
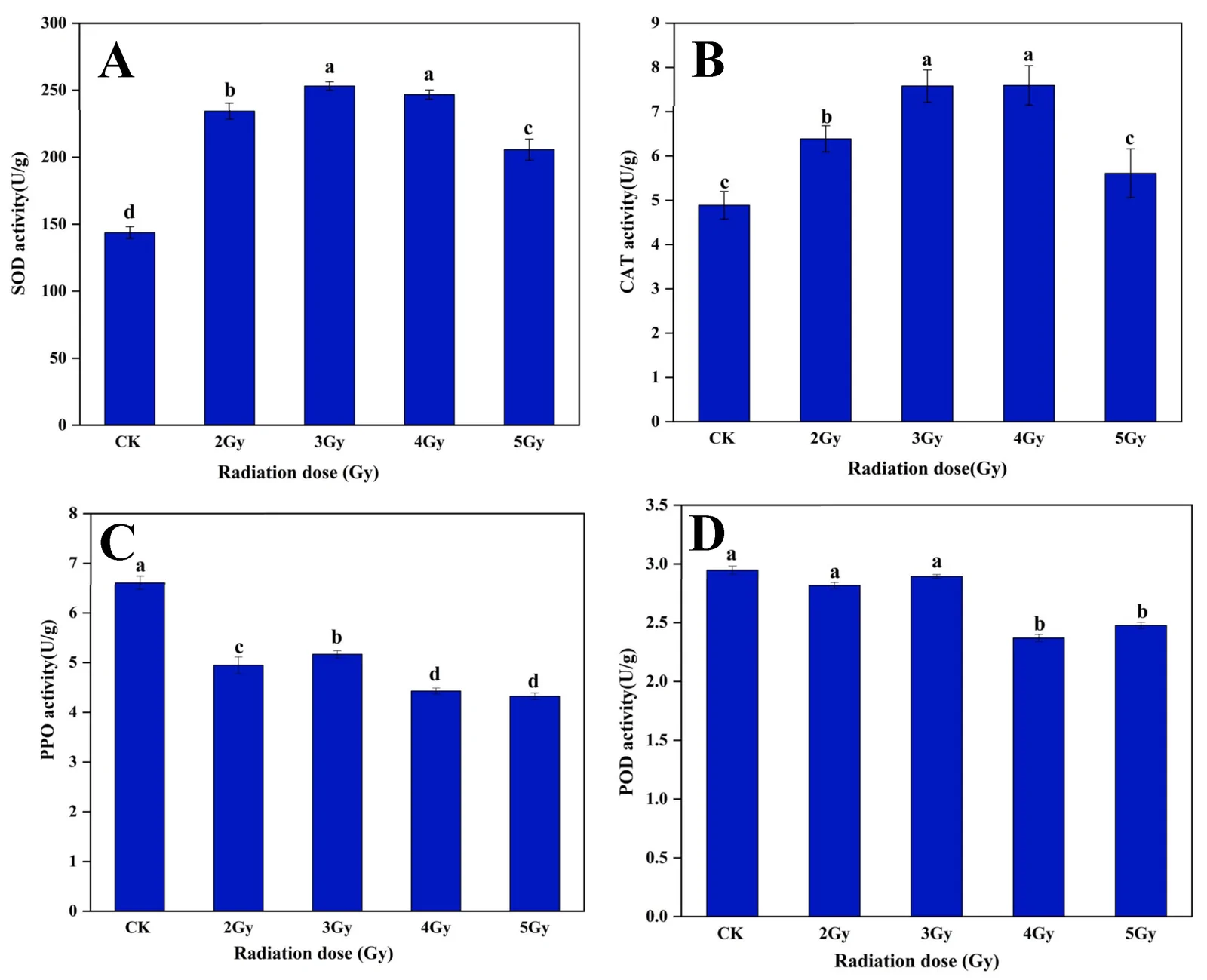
Variations in enzymatic activities of lily bulbs subjected to different doses of ^60^Co-*γ* irradiation. (**A**) SOD; (**B**) CAT; (**C**) PPO; (**D**) POD. Enzyme activities were expressed as units per gram of fresh weight (U/g FW). Different lowercase letters above the bars indicate significant differences among treatments (*p* < 0.05).

Superoxide dismutase (SOD) and catalase (CAT) constitute the first line of enzymatic defense in the plant antioxidant system [23]. Ionizing radiation triggers the generation of superoxide anions (O_2_^−^), initiating an oxidative signaling cascade [24]. SOD eliminates O_2_^−^, the precursor of reactive oxygen species (ROS), by dismutating it into H_2_O_2_ and O_2_. Subsequently, CAT decomposes high concentrations of H_2_O_2_ into water and oxygen [25]. As depicted in Figure 3A,B, irradiated lily bulbs exhibited significantly elevated SOD and CAT activities, particularly in the 3–4 Gy range (1.55–1.86 times higher than CK). These findings are consistent with previous studies [6,7,26], suggesting that low-dose radiation upregulates genes and enzymes related to antioxidant activity. This upregulation indicates that the treatment successfully activated the innate defense machinery to scavenge ROS, thereby maintaining cellular homeostasis [27,28].

PPO and POD can catalyze the oxidation of phenolic compounds. Under irradiation conditions, the oxidative stress responses of PPO and POD are as follows: PPO participates in metabolic responses, while POD not only participates in metabolic responses but also scavenges H_2_O_2_, similar to the function of the CAT enzyme [6,29]. Conversely, polyphenol oxidase (PPO) and peroxidase (POD) displayed distinct response patterns (Figure 3C,D). PPO activity was significantly inhibited under 2–5 Gy irradiation (*p* < 0.05). Since PPO is the key enzyme responsible for enzymatic browning [30], its inhibition suggests that this green processing strategy not only enhances bioactivity but also assists in preserving the visual quality of the raw material. POD activity remained stable at lower doses (2–3 Gy) but was inhibited at 4–5 Gy, likely due to inactivation by accumulated H_2_O_2_ [31]. These results also corresponded well to previous findings [6,7,21]. Specifically, this differential enzymatic response plays a synergistic role in post-harvest quality maintenance. On the one hand, the significant upregulation of SOD and CAT activities mitigates ROS-induced lipid peroxidation and cellular damage, which is crucial for delaying tissue senescence and maintaining membrane integrity [27,28]. On the other hand, the concurrent suppression of PPO and POD prevents enzymatic browning and reduces the oxidative degradation of phenolic compounds [29,31]. Consequently, the simultaneous enhancement of ROS scavengers and inhibition of browning-related enzymes demonstrates the efficacy of low-dose irradiation in preserving both the sensory attributes and nutritional value of fresh lily bulbs.

### 3.3. Metabolic Reprogramming Induced by ^60^Co-γ Irradiation

**Figure 4:**
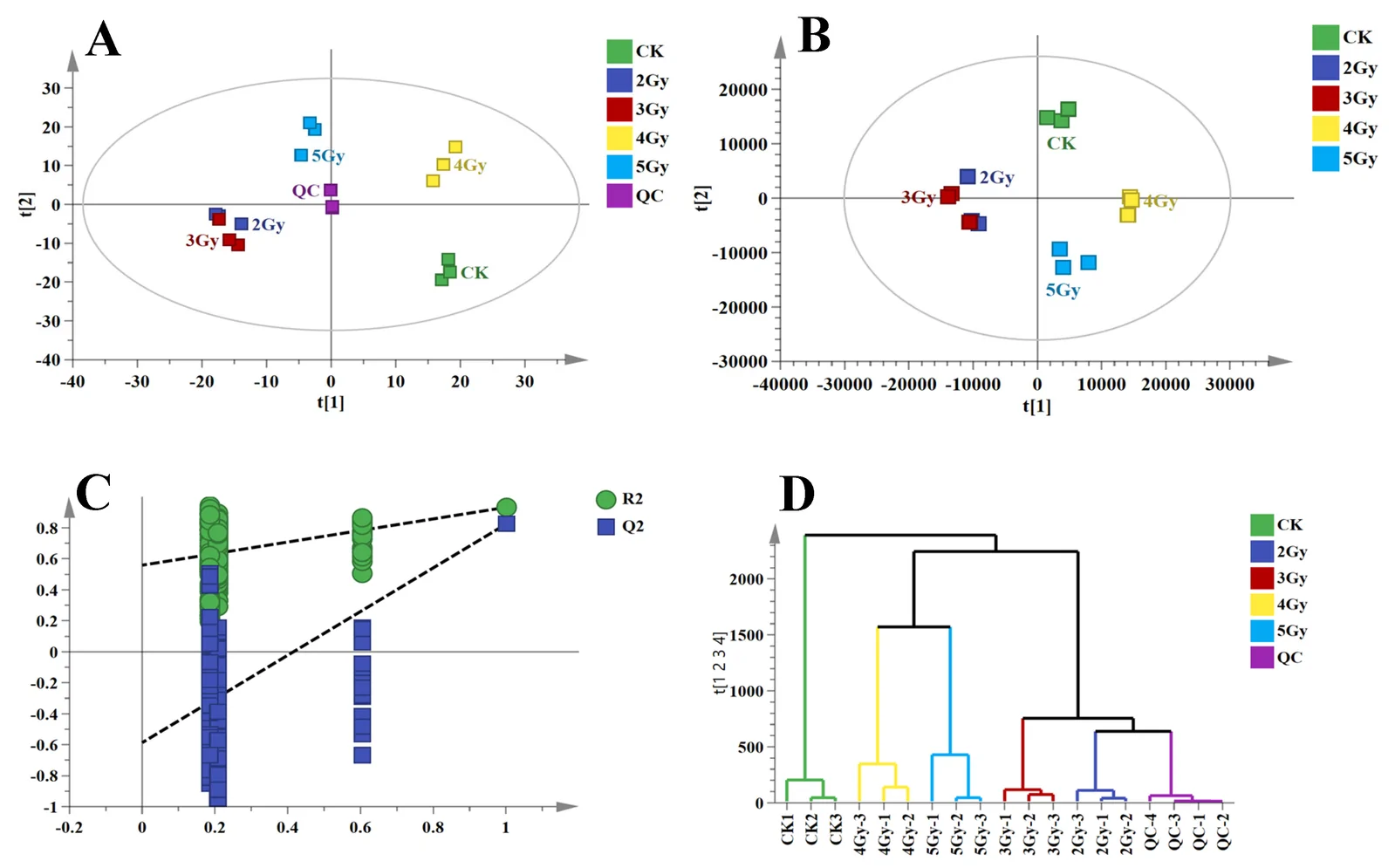
Multivariate statistical analysis of metabolic profiles in lily bulbs treated with varying irradiation doses (CK, 2, 3, 4, and 5 Gy). (**A**) PCA score plot; (**B**) PLS-DA score plot, R2X = 79.1%, R2Y = 99.6%, Q2 = 98.7%; (**C**) permutation test results (*n* = 200) for the validation of the PLS-DA model; (**D**) hierarchical clustering analysis (HCA) dendrogram. The dashed lines in panel (**C**) represent the regression lines for *R*^2^ and *Q*^2^.

To elucidate the systemic metabolic shifts, untargeted metabolomic analysis was performed using UHPLC-Q-Exactive Orbitrap MS, and the representative chromatogram of the QC sample is shown in Figure S1. The clustering of QC samples at the center of the PCA score plot (Figure 4A) indicated excellent reproducibility. Furthermore, PCA and PLS-DA score plots (Figure 4A,B) revealed a distinct separation between the irradiated groups and the control, and the PLS-DA model demonstrated excellent explanatory power and predictability (*R*^2^*X* = 79.1%, *R*^2^*Y* = 99.6%, *Q*^2^ = 98.7%), confirming that irradiation induced significant metabolic reprogramming. Notably, the 2 Gy and 3 Gy groups exhibited similar metabolic profiles, suggesting comparable compositional variations at these lower doses (Figure 4A,B,D). Validated by permutation tests (*R*^2^ = 0.406, *Q*^2^ = −0.817; Figure 4C), the PLS-DA model showed no signs of overfitting. A total of 42 differential metabolites (VIP > 1; *p* < 0.05) were identified (Table S1).

**Figure 5:**
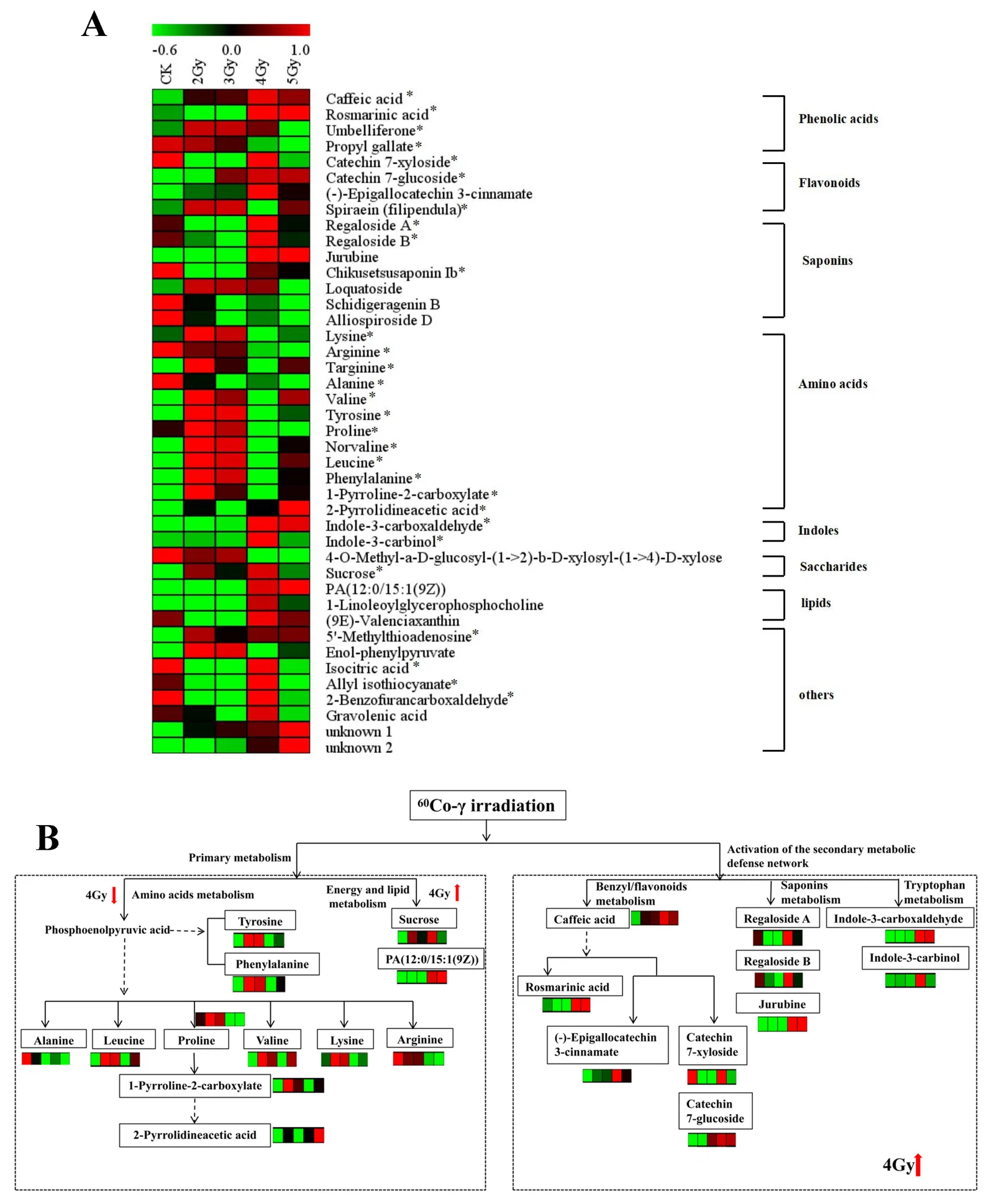
Comprehensive analysis of metabolic shifts in lily bulbs under varying irradiation doses (0, 2, 3, 4, and 5 Gy). (**A**) Heatmap visualizing the levels of differential metabolites across the five treatment groups; (**B**) schematic representation of key metabolic pathways associated with antioxidant activity. Note: An asterisk (*) indicates compounds validated by authentic standards. Data represent the mean of three biological replicates. In both panels (**A**,**B**), red and green colors denote upregulated and downregulated metabolites, respectively.

Heatmap analysis (Figure 5A) revealed three distinct metabolic response patterns: (1) The “4 Gy peak” pattern: Key antioxidants and defense signaling molecules, including caffeic acid, catechin 7-xyloside, (-)-epigallocatechin 3-cinnamate, regaloside A/B, indole-3-carboxaldehyde, and indole-3-carbinol, peaked at 4 Gy. This confirms 4 Gy as the critical dose for maximizing secondary metabolite synthesis. (2) The “4 Gy/5 Gy dual-high” pattern: Metabolites such as rosmarinic acid, jurubine (a newly detected alkaloid), and PA (12:0/15:1(9Z)) were significantly upregulated at higher doses. (3) The “2 Gy/3 Gy dual-high” pattern: Nearly all amino acids (e.g., lysine, valine, tyrosine, phenylalanine, proline) exhibited peak concentrations in the 2 Gy/3 Gy groups, suggesting that lower-dose irradiation primarily activated the reserves of primary metabolic precursors.

KEGG pathway analysis of the major differential metabolites (Figure 5B) indicated that ^60^Co-*γ* irradiation primarily activated pathways related to amino acid metabolism, energy and lipid metabolism, phenylpropanoid/flavonoid biosynthesis, saponin metabolism, and tryptophan metabolism.

Amino acids play dual roles as protein constituents and precursors for secondary metabolites [32,33]. Notably, tyrosine and phenylalanine, the initial substrates for the phenylpropanoid pathway, exhibited elevated levels in the 2 Gy/3 Gy groups. This accumulation suggests a “start-up effect,” where low-dose irradiation expands the pool of precursors necessary for subsequent phenolic synthesis. Similarly, proline, a well-known osmotic regulator and ROS scavenger [34,35], also peaked at 2–3 Gy, indicating an early adaptive response to oxidative stress.

Energy and signaling molecules showed distinct patterns. Sucrose content peaked in the 4 Gy group; beyond its role as an energy source, sucrose likely functions as a signaling molecule to regulate antioxidant gene expression during stress adaptation [36]. Meanwhile, PA (12:0/15:1(9Z)) accumulated in the 4 Gy/5 Gy groups. As a key lipid signaling molecule, this increase in phosphatidic acid may reflect membrane lipid remodeling or active repair signaling in response to higher-dose irradiation [37].

The phenylpropanoid/flavonoid pathway is the central route for plant antioxidant synthesis [38,39]. As shown in Figure 5B, the 4 Gy dose marked a physiological turning point. While upstream precursors accumulated at lower doses, the 4 Gy treatment effectively “unlocked” the downstream metabolic flux, leading to the peak accumulation of core antioxidants. Caffeic acid, a key intermediate, serves as a hub for synthesizing complex phenolics [40,41]. Consequently, downstream bioactive compounds, including rosmarinic acid, catechin-7-xyloside, and (-)-epigallocatechin-3-cinnamate, showed significant increases at 4 Gy. These complex polyphenols typically exhibit superior stability and radical scavenging capacity compared to simple phenolic acids [42,43].

Saponins and alkaloids also responded specifically to the 4 Gy threshold. Regaloside A and B, the characteristic saponins of lily bulbs with proven antioxidant activity [3,44], reached their highest levels in the 4 Gy group. Furthermore, jurubine, a steroidal alkaloid, significantly increased under 4 Gy/5 Gy irradiation. Given its molecular structure containing five hydroxyl groups, we postulate that jurubine contributes to the antioxidant capacity, serving as a novel radiation-responsive bioactive compound. Additionally, indole derivatives (indole-3-carbinol and indole-3-carboxaldehyde), which act as defense signaling molecules [45,46,47], also peaked at 4 Gy.

In summary, based on the metabolic trajectory from precursor accumulation (at 2–3 Gy) to the synthesis of complex antioxidants (at 4 Gy), we identified 4 Gy as the critical physiological threshold. This dose successfully transforms irradiation stress signals into a potent driving force for phytochemical enrichment. Future studies utilizing transcriptomics could further elucidate the expression patterns of key biosynthetic genes (e.g., *PAL*, *C4H*, *4CL*, *CHS*, *FLS*) to validate how the 4 Gy dose regulates this metabolic flux at the transcriptional level.

### 3.4. Correlation Between Metabolites and Antioxidant Capacity

To identify the specific chemical contributors to the radiation-induced antioxidant enhancement, Spearman correlation analysis was conducted between the differential metabolites and antioxidant assays (·OH, DPPH, T-AOC, and ABTS). This analysis highlighted 18 metabolites that exhibited significant positive correlations with antioxidant capacity. A deeper analysis of these 18 positively correlated metabolites reveals that they predominantly belong to distinct chemical classes, primarily including phenolic acids, flavonoids, amino acids, and alkaloids. The simultaneous positive correlation of these diverse compounds with antioxidant assays suggests that radiation treatment does not merely activate a single, isolated antioxidant pathway. Instead, it triggers a comprehensive, multi-targeted metabolic response. Phenolics and flavonoids act as direct reactive oxygen species (ROS) scavengers, while certain amino acids may serve as stress-responsive osmolytes or precursors for secondary metabolism. Together, these upregulated metabolites form a synergistic defense network to counteract radiation-induced oxidative stress.

**Figure 6:**
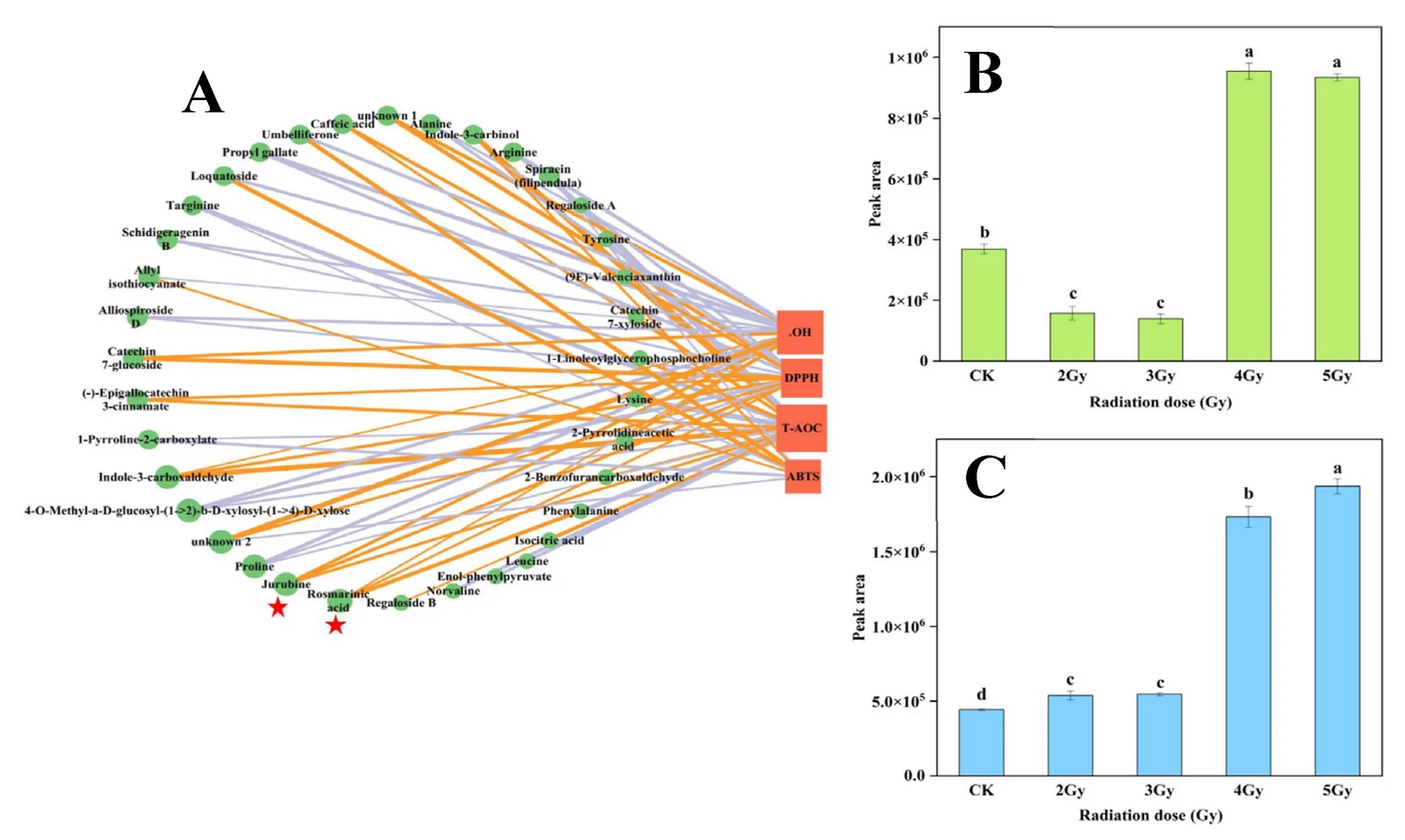
Correlation analysis between key metabolites and antioxidant capacities. (**A**) Correlation network plot. Red squares represent antioxidant indices (·OH, DPPH, T-AOC, and ABTS), and green circles represent differential metabolites. Yellow and gray lines denote positive and negative correlations, respectively. The line thickness corresponds to the magnitude of the correlation coefficient. (**B**) Variations in rosmarinic acid content under different irradiation doses; (**C**) variations in jurubine content under different irradiation doses. The red stars in panel (**A**) highlight the key metabolites (rosmarinic acid and jurubine) that are specifically analyzed in panels (**B**,**C**). Different lowercase letters above the bars indicate significant differences among treatments (*p* < 0.05).

As shown in Figure 6A, the correlation network was constructed based on connectivity degrees. The top-ranked metabolites emerging as key contributors included rosmarinic acid, jurubine, indole-3-carboxaldehyde, (-)-epigallocatechin 3-cinnamate, catechin 7-glucoside, and one unidentified compound (Unknown2). Specifically, rosmarinic acid demonstrated robust correlations with T-AOC (r = 0.73, *p* < 0.01), ·OH (r = 0.53, *p* < 0.05), and DPPH scavenging activities (r = 0.58, *p* < 0.05). Similarly, indole-3-carboxaldehyde showed a particularly strong association with T-AOC (r = 0.86, *p* < 0.01). Notably, well-established antioxidants in lily bulbs, such as caffeic acid and regaloside A/B, also displayed significant positive correlations with T-AOC (r > 0.60) and DPPH, corroborating their role in the basal antioxidant defense system. However, the radiation-induced upregulation of other potent compounds suggests the recruitment of a broader defense network.

Rosmarinic acid, a polyphenolic compound, is renowned for its superior antioxidant efficacy, often surpassing that of vitamin E and chlorogenic acid [48,49]. While not traditionally considered a primary characteristic component of lily bulbs (unlike regaloside A/B), our results indicate that its levels significantly increased in the 4 Gy/5 Gy groups (Figure 6B). Consistent with previous reports of trace rosmarinic acid in lilies [50,51], our findings suggest that low-dose irradiation effectively “awakens” the biosynthesis of this potent polyphenol, allowing it to contribute disproportionately to the overall bioactivity despite its lower abundance relative to major saponins.

Crucially, we report the detection of jurubine in lily bulbs (*Lilium lancifolium* Thunb.) for the first time. As shown in Figure 6C, the content of this steroidal alkaloid was significantly upregulated in the 4 Gy/5 Gy groups compared to the control. Correlation analysis revealed that jurubine was strongly associated with ·OH (r = 0.67, *p* < 0.01), T-AOC (r = 0.63, *p* < 0.05), and DPPH (r = 0.66, *p* < 0.01) scavenging capacities. This aligns with prior studies indicating that steroidal alkaloids possess structural features capable of radical scavenging [52,53,54]. The discovery of jurubine as a radiation-responsive metabolite expands the known phytochemical profile of edible lilies and highlights steroidal alkaloids as a potentially overlooked class of antioxidants in this species.

### 3.5. Molecular Docking of Rosmarinic Acid and Jurubine with DPPH

To provide mechanistic insights into the observed antioxidant enhancement, molecular docking was employed to investigate the interactions of rosmarinic acid and jurubine with the DPPH radical. Given that these two metabolites showed the most significant correlations with antioxidant assays (•OH, T-AOC, and DPPH), determining their binding modes is crucial for understanding the chemical basis of their bioactivity [44,55,56].

**Figure 7:**
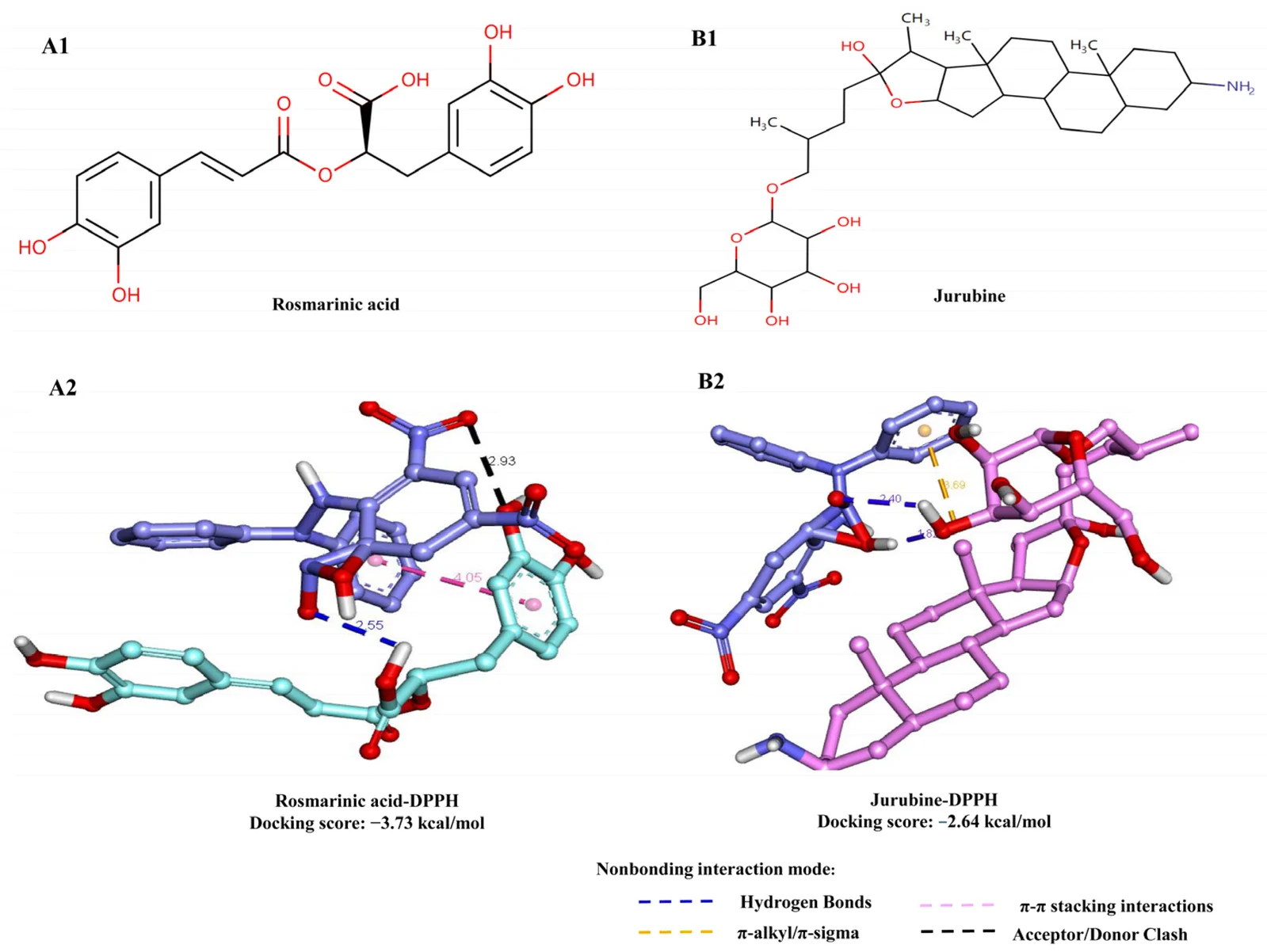
Molecular docking analysis elucidating the antioxidant mechanisms of rosmarinic acid and jurubine. (**A1**) Chemical structure of rosmarinic acid; (**A2**) molecular docking simulation of rosmarinic acid with the DPPH radical; (**B1**) chemical structure of jurubine; (**B2**) molecular docking simulation of jurubine with the DPPH radical. In the 3D docking models (**A2**, **B2**), the DPPH radicals are displayed as slate blue stick models, while rosmarinic acid and jurubine are colored cyan and magenta, respectively.

Rosmarinic acid (Figure 7(A1)) is a well-established antioxidant characterized by its multiple hydroxyl groups and conjugated double bonds [48,49]. The docking simulation (Figure 7(A2)) revealed a robust binding affinity of −3.73 kcal/mol with the DPPH radical. The interaction was primarily stabilized by hydrogen bonds and π-π stacking interactions, indicating the formation of a stable complex that facilitates subsequent radical scavenging. It is worth noting that alongside the favorable interactions, molecular docking also predicted a localized acceptor/donor clash (unfavorable electrostatic repulsion) in the complex (Figure 7A2); however, the overall docking score (−3.73 kcal/mol) remains negative. This indicates that the strong energy contributions from the extensive hydrogen bonding and π-interactions sufficiently overcome the local destabilizing penalty caused by the clash, maintaining the overall thermodynamic stability of the simulated complexes.

Jurubine (Figure 7(B1)), the steroidal alkaloid identified in this study, exhibited a theoretical binding energy of −2.64 kcal/mol, indicating a relatively weak and transient interaction, which can be attributed to the bulky steroidal skeleton of jurubine that hinders deep encapsulation with the DPPH radical. The docking analysis (Figure 7(B2)) showed that it can engage with DPPH via hydrogen bonds and π-alkyl/π-sigma contacts. Since a commercial reference standard for jurubine is currently unavailable, direct in vitro validation was not feasible. In this context, molecular docking serves as a preliminary predictive tool. These theoretical results indicate that jurubine represents a radiation-responsive potential candidate, whose actual antioxidant contribution and mechanism require future targeted isolation and empirical verification.

In summary, this computational analysis elucidates the spatial and electrostatic complementarity between these metabolites and free radicals. While molecular docking primarily simulates the thermodynamic stability of the pre-reaction complex rather than the full reaction kinetics [57,58,59], the identification of specific binding modes offers compelling structural evidence supporting our hypothesis: the radiation-induced accumulation of rosmarinic acid and jurubine contributes directly to the enhanced bioactivity of lily bulbs via effective radical binding.

### 3.6. Practical Implications and Industrial Feasibility

From a practical application perspective, it should be noted that the implementation of ^60^Co-*γ* irradiation requires highly specialized and strictly regulated centralized facilities, which limits its on-site application in small-scale farms. However, food irradiation is already a mature and commercialized post-harvest technology globally, officially approved by organizations such as the Food and Agriculture Organization of the United Nations (FAO), the World Health Organization (WHO), and the U.S. Food and Drug Administration (FDA). Therefore, leveraging existing regional industrial irradiation centers offers a highly feasible strategy. Ultimately, our findings provide a novel “value-added” application for this existing industrial infrastructure, demonstrating that low-dose irradiation can serve as an effective abiotic elicitor to pre-enrich beneficial phytochemicals prior to further processing (e.g., freeze-drying), thereby maximizing the functional and commercial value of agricultural products.

## 4. Conclusions

This study successfully validates a novel, green, and energy-efficient post-harvest metabolic enhancement strategy for lily bulbs (*Lilium lancifolium* Thunb.) by utilizing low-dose ^60^Co-*γ* irradiation (2–5 Gy) coupled with immediate freeze-drying. Our findings demonstrate that 4 Gy serves as a critical physiological threshold, effectively activating innate antioxidant defense mechanisms via the hormesis effect. This activation was evidenced by the significant enhancement of SOD and CAT enzymatic activities (increasing by 1.55- to 1.86-fold), as well as the substantial accumulation of total phenolics and flavonoids (which peaked at 1.31- and 2.69-fold higher than the control, respectively). Furthermore, untargeted metabolomic analysis identified 42 differential metabolites, revealing that the 4 Gy dose primarily upregulated the phenylpropanoid and flavonoid biosynthetic pathways. This metabolic reprogramming promoted the synthesis of potent antioxidants, including rosmarinic acid, caffeic acid, and regaloside A/B. Notably, we report for the first time the detection of the steroidal alkaloid jurubine in lily bulbs, identifying it as a novel radiation-responsive metabolite. Integrated correlation analysis (e.g., positive correlations with DPPH at r = 0.66 for jurubine and r = 0.58 for rosmarinic acid) and molecular docking (yielding binding energies of −2.64 kcal/mol and −3.73 kcal/mol against DPPH, respectively) simulations offered computational predictions regarding the theoretical interactions of jurubine and rosmarinic acid with free radicals (e.g., DPPH), suggesting they may act as potential candidates contributing to the observed antioxidant enhancement. Collectively, these results offer new insights into the stress response mechanisms of lily bulbs and confirm that controlled ^60^Co-*γ* irradiation (specifically at 4 Gy) is a promising post-harvest processing strategy. Moving forward, future studies should focus on the targeted extraction, purification, and in vivo biological validation of these key radiation-induced metabolites (particularly jurubine and rosmarinic acid) to further elucidate their precise in vivo antioxidant mechanisms. By transforming abiotic stress into a driving force for nutrient accumulation, this work provides a theoretical basis and a sustainable technical route for the industrial production of superior, antioxidant-enriched lily-based functional food ingredients.

## Data Availability

The original contributions presented in this study are included in the article and Supplementary Materials. Further inquiries can be directed to the corresponding authors.

## Supplementary Materials

The following supporting information can be downloaded at: https://www.mdpi.com/article/10.3390/antiox15091071/s1, Table S1. Summary of putatively identified differential metabolites in lily bulbs (*Lilium lancifolium* Thunb.). Figure S1. Representative total ion chromatogram (TIC) of a quality control (QC) sample for bulb metabolomic analysis.
