# Supplementary material for: Green and Energy-Efficient Post-Harvest Enhancement: Low-Dose 60Co-γ Irradiation Enhances the Bioactive Profile of Lily Bulbs (Lilium lancifolium Thunb.) as Functional Food Ingredients
Source: Antioxidants (Basel). 2026 Aug 26;15(9):1071. doi: 10.3390/antiox15091071 (PMC13603400; doi:10.3390/antiox15091071)
Supplement: Supplementary file 1 [file antioxidants-15-01071-s001.zip › antioxidants-4387074-supplementary.pdf]

Green and energy-efficient biofortification: Low-dose ^{60}Co-*γ* irradiation enhances the bioactive profile of lily bulbs (*Lilium lancifolium* Thunb.) as functional food ingredients

Yao Hu[a], Lihui Li[a], Xingyu Lei[a], Yiji Zhou[a], Wei Gong[a], Mengshan Sun[a, *], Rong Song[a, **]

[a] Hunan Institute of Nuclear Agriculture Sciences and Chinese Herbal Medicines, Hunan Academy of Agricultural Sciences, Changsha, Hunan 410125, China

Corresponding Author

* Corresponding author: Email:sunmengshan@hunaas.cn

**Corresponding author: Email: songrong0205@126.com

Purchase information of standards

Caffeic acid, Rosmarinic acid, Umbelliferone, Propyl gallate, Catechin 7-xyloside, Catechin 7-glucoside, Regaloside A, Regaloside B, Jurubine, Chikusetsusaponin Ib, Lysine, Arginine, Targinine, Alanine, Valine, Tyrosine, Proline, Norvaline, Leucine, Phenylalanine, 1-Pyrroline-2-carboxylate, 2-Pyrrolidineacetic acid, Indole-3-carboxaldehyde, Indole-3-carbinol, Sucrose, 5'-Methylthioadenosine, Isocitric acid, Allyl isothiocyanate, and 2-Benzofurancarboxaldehyde were obtained from Sigma (St. Louis, MO, USA).

Raw data analysis

Raw metabolomic data acquired from UHPLC−QTOF/MS were first treated for peak picking using MassHunter Qualitative Analysis software (B.07.00 SP1, Agilent Technologies, Santa Clara, CA, USA) and then for peak alignment using Mass Profiler Professional (version 13.0, Agilent Technologies, Santa Clara, CA, USA). The signal-to-noise (S/N) threshold for peak detection was set to 3. The retention time tolerance and mass tolerance for the peak alignment was set to 0.2 min and 0.01 Da, respectively. The obtained compound ion features with relative standard deviations (RSD) of mass intensities less than 30% in QC samples were used for multivariate and univariate analysis. Compounds were structurally identified based on authentic standards, MS^2 spectra, mass accuracy, and metabolomic databases (Metlin and HMDB).

Table S1. Summary of putatively identified differential metabolites in lily bulbs (*Lilium lancifolium* Thunb.).

Compound identification	RT (min)	Accurate mass (m/z)	Theoretical mass (ESI+)	Mass error (ppm)	Ionization model	Formula	Identified by
**Phenolic acids**							
Caffeic acid	12.67	163.0391	163.0395	-2.4534	$[M - H_2O + H]^+$	$C_9H_8O_4$	Standard
Rosmarinic acid	9.83	361.0894	361.0923	-8.0312	$[M + H]^+$	$C_{18}H_{16}O_8$	Standard
Umbelliferone	7.74	163.0392	163.0395	-1.8400	$[M + H]^+$	$C_9H_6O_3$	Standard
Propyl gallate	0.60	213.0749	213.0763	-6.5704	$[M + H]^+$	$C_{10}H_{12}O_5$	Standard
**Flavonoids**							
Catechin 7-xyloside	9.22	423.1262	423.1291	-6.8537	$[M + H]^+$	$C_{20}H_{22}O_{10}$	Standard
Catechin 7-glucoside	11.52	453.1368	453.1397	-6.3998	$[M + H]^+$	$C_{21}H_{24}O_{11}$	Standard
(-)-Epigallocatechin 3-cinnamate	18.80	437.1207	437.1236	-6.6343	$[M + H]^+$	$C_{24}H_{20}O_8$	MS^2, database
Spiraein (filipendula)	7.72	439.1210	439.1211	-2.2772	$[M + Na]^+$	$C_{18}H_{24}O_{11}$	MS^2, database
**Saponins**							
Regaloside A	10.55	443.1318	443.1317	+0.0756	$[M+Na]^+$	$C_{18}H_{24}O_{10}$	Standard
Regaloside B	13.90	465.1369	465.1372	-0.8132	$[M + Na]^+$	$C_{20}H_{26}O_{11}$	Standard
Jurubine	14.67	578.4011	578.4056	-7.7800	$[M - H_2O + H]^+$	$C_{33}H_{57}NO_8$	Standard
Chikusetsusaponin Ib	17.23	927.4918	927.4953	-3.7736	$[M + H]^+$	$C_{47}H_{74}O_{18}$	Standard
Loquatoside	8.98	439.1211	439.1240	-6.6041	$[M + H]^+$	$C_{20}H_{22}O_{11}$	MS^2, database
Schidigeragenin B	19.98	429.2999	429.3005	-1.3976	$[M + H]^+$	$C_{27}H_{40}O_4$	MS^2, database
Alliospiroside D	19.97	755.4212	755.4218	-7.9425	$[M + H]^+$	$C_{39}H_{62}O_{14}$	MS^2, database
**Amino acids**							
Lysine	0.57	147.1129	147.1133	-2.7190	$[M + H]^+$	$C_6H_{14}N_2O_2$	Standard
Arginine	0.62	175.1190	175.1195	-2.8552	$[M + H]^+$	$C_6H_{14}N_4O_2$	Standard

Targinine	0.64	189.1347	189.1351	-2.1148	$[M + H]^+$	$C_7H_{16}N_4O_2$	Standard
Alanine	0.65	90.0554	90.101	-0.0005	$[M + H]^+$	$C_3H_7NO_2$	Standard
Valine	0.68	118.0865	118.0868	-2.5405	$[M + H]^+$	$C_5H_{11}NO_2$	Standard
Tyrosine	0.79	182.0814	182.0817	-1.6476	$[M + H]^+$	$C_9H_{11}NO_3$	Standard
Proline	0.71	116.0709	116.0711	-1.7230	$[M + H]^+$	$C_5H_9NO_2$	Standard
Norvaline	1.05	118.0866	118.0867	-8.4683	$[M + H]^+$	$C_5H_{11}NO_2$	Standard
Leucine	1.46	132.1021	132.1024	-2.2709	$[M + H]^+$	$C_6H_{13}NO_2$	Standard
Phenylalanine	3.13	166.0865	166.0867	-1.2041	$[M + H]^+$	$C_9H_{11}NO_2$	Standard
1-Pyrroline-2-carboxylate	0.73	114.0553	114.0555	-1.7535	$[M + H]^+$	$C_5H_7NO_2$	Standard
2-Pyrrolidineacetic acid	0.65	130.0864	131.0731	-0.0075	$[M + H]^+$	$C_6H_{11}NO_2$	Standard
**Indoles**							
Indole-3-carboxaldehyde	5.38	146.0602	146.0606	-2.7386	$[M + H]^+$	C_9H_7NO	Standard
Indole-3-carbinol	12.44	130.0653	130.0656	-2.3065	$[M - H_2O + H]^+$	C_9H_9NO	Standard
**Saccharides**							
4-O-Methyl-a-D-glucosyl-(1->2)-b-D-xylosyl-(1->4)-D-xylose	0.69	490.1768	490.1772	-0.81603	$[M + NH_4]^+$	$C_{17}H_{28}O_{15}$	MS^2, database
Sucrose	0.76	365.1054	365.1060	-1.6434	$[M + Na]^+$	$C_{12}H_{22}O_{11}$	Standard
**Lipids**							
PA(12:0/15:1(9Z))	14.23	577.3930	577.3869	1.0564	$[M + H]^+$	$C_{34}H_{65}O_8P$	MS^2, database
1-Linoleoylglycerophosphocholine	21.08	520.3400	520.3403	-5.7654	$[M + H]^+$	$C_{26}H_{50}NO_7P$	MS^2, database
(9E)-Valenciaxanthin	20.63	413.3051	413.3055	-9.6780	$[M + H]^+$	$C_{27}H_{40}O_3$	MS^2, database
**others**							
5'-Methylthioadenosine	6.02	298.0969	298.0974	-1.6773	$[M + H]^+$	$C_{11}H_{15}N_5O_3S$	Standard
Enol-phenylpyruvate	1.48	182.0815	182.0817	-1.0984	$[M + NH_4]^+$	$C_9H_8O_3$	MS^2, database
Isocitric acid	0.79	215.0163	215.0168	-2.3254	$[M + Na]^+$	$C_6H_8O_7$	Standard

Allyl isothiocyanate	0.53	100.0248	100.02207	2.7293	$[M + H]^+$	C_4H_5NS	Standard
2-Benzofurancarboxaldehyde	9.22	147.044 2	147.0446	-2.7202	$[M + H]^+$	$C_9H_6O_2$	Standard
Gravolenic acid	13.89	281.1019	281.1025	-2.1344	$[M + H]^+$	$C_{14}H_{16}O_6$	MS^2, database
unknown 1	1.799	205.0085	-		-	-	-
unknown 2	18.51	407.1099	-		-	-	-

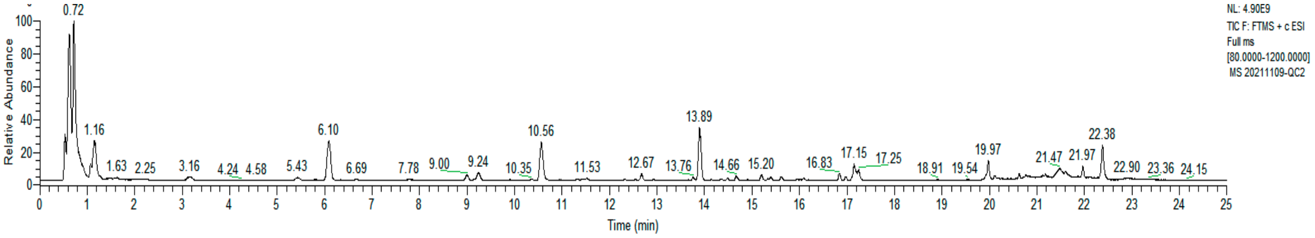

Figure S1. Representative total ion chromatogram (TIC) of a quality control (QC) sample for bulb metabolomic analysis.
